# ORTHOSTATIC SUPPORT IN PARAPLEGIC AND AMPUTEE PATIENTS: A CONTROLLED TRIAL

**DOI:** 10.1590/1413-785220243201e271849

**Published:** 2024-03-22

**Authors:** Gisele Harumi Hotta, Débora Pinheiro Aguiar, Gabriella Coelho Vieira de Melo Alves, Liana Praça Oliveira, Marie Aquino Melo de Leopoldino, Jefferson Pacheco Amaral Fortes, Francisco Carlos de Mattos Brito Oliveira, Francisco Fleury Uchoa Santos

**Affiliations:** 1 Universidade de São Paulo, School of Medicine of Ribeirão Preto, Health Sciences Department, Ribeirão Preto, SP, Brazil.; 2 Centro de Pesquisa, Desenvolvimento e Inovação da Dell, Dell Lead, Fortaleza, CE, Brazil.; 3 Centro Universitário Estácio do Ceará, Physiotherapy Department, Fortaleza, Brazil.; 4 Universidade Estadual do Ceará, Computation Sciences Department, Fortaleza, CE, Brazil.

**Keywords:** Spinal Cord Injuries, Amputees, Posture, Traumatismos da Medula Espinal, Amputados, Postura

## Abstract

**Introduction:**

Functional incapacity caused by physical alterations leads to significant limitations in daily activities and has a major impact on the return of people with disabilities to the social space and the workplace. This calls for an evaluation of the long-term influence of the use of a device specially developed for orthostatic posture on the physiological, biomechanical and functional parameters of amputees and spinal cord patients.

**Objective:**

The objective was evaluate the effect of postural support device use on function, pain, and biomechanical and cardiologic parameters in spinal cord injury and amputees patients compared to a control group.

**Methods:**

The orthostatic device was used by the participants for a period of ten consecutive days, for three cycles of 50 minutes each day, and a 15-day follow-up. Participants were positioned and stabilized using adjustable straps on the shoulders, trunk, and hips. The primary outcome was brief pain inventory. Fifteen participants were included the control group, 15 in the amputee group, and 15 in the spinal cord group.

**Results:**

Our results demonstrate that the use of the device allows the orthostatic position of amputees and spinal cord patients evaluated for ten days, leading to improved functionality and pain in the spinal cord and amputee groups compared to the control group. In addition, no changes were observed for secondary outcomes, indicating that the use of the device did not cause harm interference to patients.

**Conclusion:**

The long-term use of the orthostatic device is beneficial for improving functionality, reduce pain in amputees and spinal cord injury patients. ***Level of evidence II; Therapeutic Studies - Investigating the results of treatment***.

## RESUMO

**Introdução:**

A incapacidade funcional causada por alterações físicas leva a limitações significativas nas atividades diárias e gera um grande impacto no retorno das pessoas com deficiência ao espaço social e ao local de trabalho, demandando a avaliação da influência em longo prazo do uso de um dispositivo especialmente desenvolvido para a postura ortostática nos parâmetros fisiológicos, biomecânicos e funcionais de pacientes amputados e com medula espinhal.

**Objetivo:**

O objetivo foi avaliar o efeito do uso do dispositivo de suporte postural na função, dor e parâmetros biomecânicos e cardiológicos em pacientes com lesão medular e amputados em comparação com um grupo controle.

**Métodos:**

O aparelho ortostático foi utilizado pelos participantes por um período de dez dias consecutivos, em três ciclos de 50 minutos diários, com acompanhamento de 15 dias. Os participantes foram posicionados e estabilizados por meio de alças ajustáveis nos ombros, tronco e quadris. O desfecho primário foi o questionário Breve Inventário de Dor. Quinze participantes foram incluídos no grupo controle, 15 no grupo amputado e 15 no grupo medular.

**Resultados:**

Nossos resultados demonstram que o uso do dispositivo permite a posição ortostática de amputados e pacientes com lesão medular avaliados por dez dias, levando a melhora da funcionalidade e dor nos grupos de amputados e medula espinhal em relação ao grupo controle. Além disso, não foram observadas alterações nos resultados secundários, indicando que o uso do dispositivo não causou interferência prejudicial aos pacientes.

**Conclusão:**

O uso prolongado do dispositivo ortostático é benéfico para melhorar a funcionalidade, reduzir a dor em amputados e pacientes com lesão medular. ***Nível de Evidência II; Estudos Terapêuticos - Investigação dos resultados de tratamento.***

## INTRODUCTION

Functional disability caused by physical alterations leads to significant limitations in daily activities and generates a great impact on the return of people with disabilities into the social space and workplace. Limited mobility has been associated with important psychosocial factors that lead a high risk of developing severe symptoms of depression and consequent worsening health-related quality of life.^1-3^

About 250.000 to 500.00 people worldwide suffer from spinal cord injury, which results in paralysis, sensorimotor deficit, and consequently sedentary behavior, generating direct effects on health, functioning, and functional independence.^2^ In the United States, about 1.7 million people reported the loss of a limb in 2007,^4^ with the number of lower limb amputations increasing annually due to the high incidence of diabetes and cardiovascular disease.^5^ The impact of lower limb amputation resulting from traumatic illness or injury is great for walking skills and community engagement.^6^ Considering that the number of people affected by both conditions is high and predispose to relevant factors such as limited mobility and social reintegration, inclusion strategies should be prioritized to reduce the psychosocial and physical risks associated with the dysfunction.^7^

Changes in the spinal cord lead to peripheral and central cardiovascular adaptations such as increased peripheral vascular resistance, reduced capillarization, and decreased artery diameters, which cause static hypotension resulting from a decline in blood pressure, limiting the stand position.^8^ On the other hand, amputees also show changes in heart rate due to loss of aerobic capacity related to reduced walking ability and show changes in muscle strength and balance due to deconditioning and disuse.^9^ To re-establishment of the locomotion and mobility functions of patients with physical disabilities related to the lower limb, assistive devices have been developed to allow the standing position and gait.^10,11^ Thus, the function of the orthoses is to generate stability of the affected limb and, in some cases, assist in the gait pattern with the lowest possible energy consumption.^10,11^

Ergonomic position is necessary and has been developed regarding the need for changes in work to accommodate workers’ characteristics.^12^ Considering that when returning to the work and social environment, the individual with disability needs follow to a specific workplace rules, like a specific workload and specific positions adopted in a laboral environment. That is why limitations in the use of positioning orthosis occur since they generate a very large amount of physical energy expenditure and generated limited mobility. In addition, in most cases, prior training is required to restore the patient’s aerobic capacity and strength to do the task.^8,9^ To the best of our knowledge, this is the first study that aims to assess the influence of the use of a device specially developed for the orthostatic posture on the physiological, biomechanical, and functional parameters of spinal cord and amputee patients in long term. The objective was to evaluate the effect of postural support device use on function, pain, and biomechanical and cardiologic parameters in spinal cord injury and amputees patients compared to a control group.

## METHODS

### Design

A non-randomized controlled trial. The study was approved by the Local Ethics Committee (CAAE 30603420.3.0000.5040) and prospectively registered at Registro Brasileiro de Ensaios Clínicos (REBEC) (identifier U1111-1257-5736). All participants were informed about the procedures and signed the written consent form. All methodological steps followed the recommendations of Consolidated Stands of Reporting Trials (CONSORT).

### Participants

The participants were recruited between October and December 2020 in the Centro de Pesquisa, Desenvolvimento e Inovação Dell – DELL LEAD. The eligibility criteria were: adults, aged between 18-50 years, height 1.55-1.75 cm, maximum weight 100 kg, of both sexes, without associated vascular pathologies (clotting disorders, decompensated diabetes, etc.), with stabilized blood pressure. The control group was composed by healthy people and without motor changes in the lower limbs. In the amputee group was selected people with unilateral lower-limb amputee. The paraplegic group was selected people with diagnostic of a total or partial paraplegic, with a maximum of 10 years of the lesion and without musculoskeletal deformities in lower limbs.

The exclusion criteria were: individuals with height and weight outside of the defined, established limits, unstable blood pressure, and severe vascular alterations. Furthermore, they exclude people with cognitive or psychological dysfunction that influence the performance of the tests, like panic syndrome, severe anxiety, or depression.

### Intervention

The proposed intervention consisted of using the orthostatic device by the participants for a period of ten consecutive days, used for three cycles of 50 minutes each day, with a 10-minute rest between each cycle and a 15-day follow-up after the end of the experiment. Initially, demographic data were collected and applied to the questionnaires: Brief pain Inventory (BPI),^13^ Functional Independence Measure (FIM),^14,15^ and sleep quality by Epiworth Sleepiness Scale.^16^ Then, measurements of vital signs [blood pressure (BP), oxygen saturation (SatO2), and heart rate (HR)] were performed. On a stretcher, the participants were positioned to collect measures of joint range of motion^17^ and muscle strength using a handheld dynamometer.^18^

Participants were positioned in the orthostatic device and stabilized using adjustable straps on the shoulders, trunk, and hips. In addition, a wider velcro band was added in the region of the legs (above the knee) and the region of the tibialis anterior. These bands allowed greater stability of the lower limb in the standing posture, especially for the paraplegic group, due to limitations in motor control. Once positioned, the device was elevated, allowing the participant to remain in the standing position. At the base of the equipment, a mechanism was installed that allowed movement in ankle dorsiflexion to generate mobility in the joint during the orthostatic position.

After the volunteers were placed in orthostatism, evaluations of the autonomic nervous system (heart rate variability), body thermography of the trunk, hip, thigh, leg, and feet regions, and plantar pressure by baropodometry were performed. During the proposed period of daily intervention, consisting of three cycles of 50 minutes of standing position, the variables heart rate, oxygen saturation, and blood pressure were measured every 25 minutes to monitor the stability of vital signs. After completing the three cycles of 50 minutes with a 10-minute rest (totaling 3 hours of equipment use), the heart rate variability, body thermography of the trunk, hip, thigh, knee, leg, and feet regions, and plantar pressure were measured again. The volunteers were again positioned on the stretcher, and measurements of range of motion and muscle strength were taken. The study variables were collected at baseline, on the fifth day, at the end of the experiment (tenth day), and at follow-up.

### Outcome measures

Trained therapists collected all outcome measurements. The measures were obtained before the intervention (baseline), five days, ten days (end of intervention), and 15 days after the end of intervention (follow up).

Functionality was considered the primary outcome. Secondary outcomes were: sleepiness level, pain, plantar pressure, muscle strength, ROM, oxygen saturation, heart rate variability, and thermography.

Function, Pain, and level of sleepiness was measured with Functional Independence Measure (FIM)^4,15^ and Brief Pain Inventory (BPI) was used for pain assessment.^13^ The sleepiness assessment was performed using the Epworth Sleepiness Scale^16^

The analysis of mean plantar pressure and stabilometry was performed by a baropodometer (T-Plateda Medicapteurs®, France).^19^ A FLIR C2 6.4 infrared camera was used to collect thermographic data. Averages of the trunk, hip, thigh, knee, leg, and feet regions were performed. Data were analyzed and edited by FLIR Tools+. The images were captured at a fixed distance of one meter away from the participant, and the room temperature was regulated at 25 degrees.^20^

Heart rate variability: For data collection, EmWave® software (Quantum Intech, Inc. Boulder Creek, CA, USA)^21^ was used. Muscle strength and ROM: The assessment of muscle strength was performed using a manual dynamometer (SP Tech, Medeor MedTech, Santa Catarina, Brazil), with a maximum capacity of 90.72 kgf (200 lbf) and reliable for use in this population.^22^ Three isometric contractions lasting 15 seconds were performed for trunk extension, knee flexion and extension, internal and external rotation of the hip and and plantar flexion and dorsiflexion of the ankle (Supplemental Material).

### Statistical analysis

Statistical analysis followed the intention-to-treat concept and was carried out by a researcher not involved in the evaluation and treatment protocols. A significance level of 0.05 was set. The linear mixed-effect model was applied to the primary and secondary variables. "Time" and "group" were considered fixed effects, whereas the participants were considered the random effect. The time by group interaction was included in the analysis to assess the differential effect between the groups at each follow-up. The dependent variable baseline value was included as a covariate to correct possible differences. Statistical analyses were performed using the SPSS.

## RESULTS

**Figure 1:**
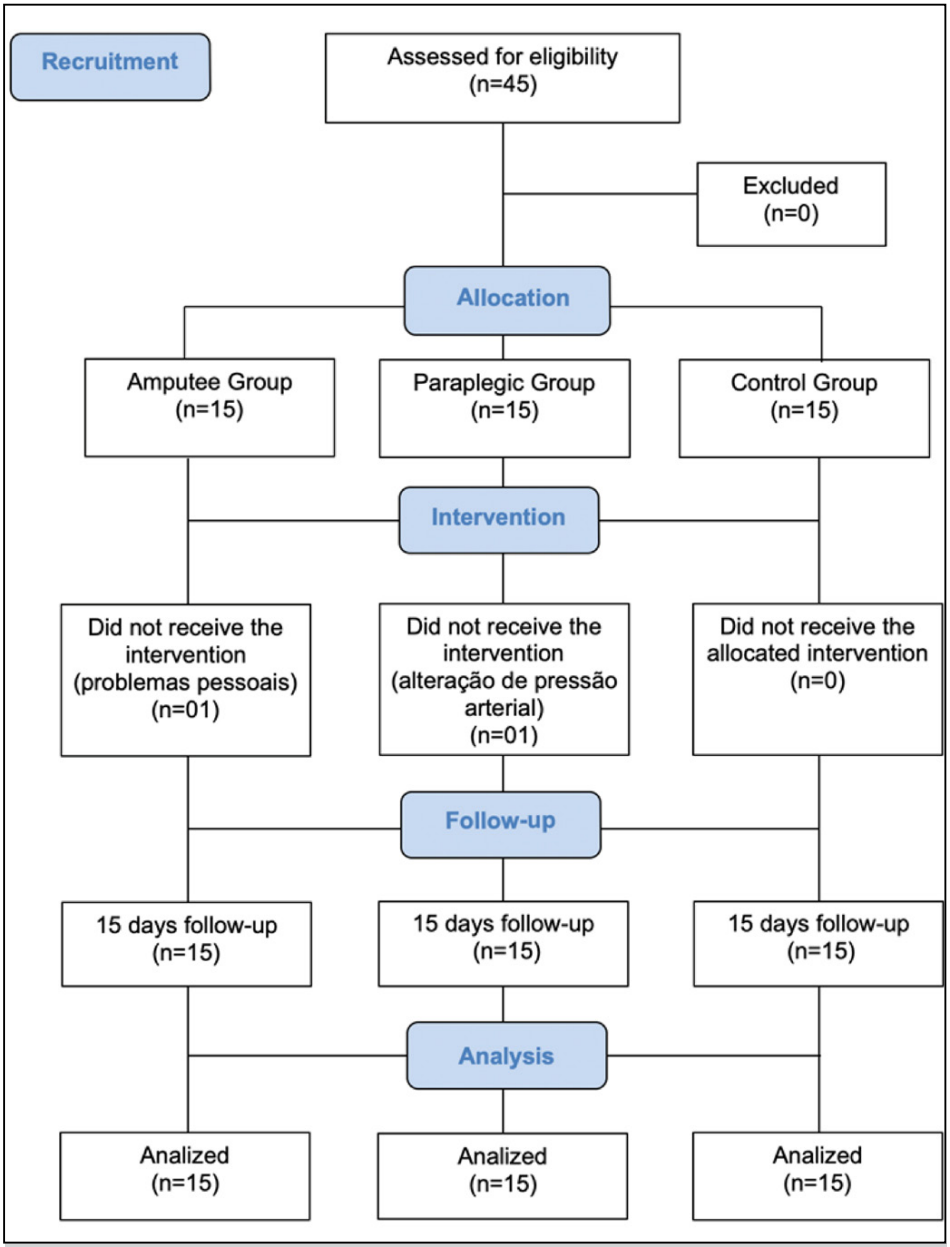
Flow chart of participant inclusion criteria.

Forty five participants were included: 15 in the control group, 15 in the amputee group, and 15 in the paraplegic group. The participants’ baseline characteristics are presented in Table 1. One individual in the amputee group dropped out for personal reasons and one in the paraplegic group dropped out for blood pressure decompensation. (Figure 1)

**Table 1:** Sociodemographic characteristics of the study sample (n=45).

		Control (N=15)			Amputees (N=15)			Spinal Cord (N=15)	
Sex									
Male		4			9			12	
Female		11			6			3	
**Marital status**									
Not married		12			10			11	
Married		3			5			4	
**Physical activity**									
practitioners		8			14			11	
non-practitioners		7			1			4	
	**Average**		**SD**	**Average**		**SD**	**Average**		**SD**
Age (years)	22.73		2.54	35.00		8.66	31.4		7.80
height (m)	1.63		0.09	1.65		0.07	1.68		0.10
Weight (kg)	67.85		14.72	73.26		12.67	62.13		14.32
BMI (kg/m ^2^)	25.51		4.69	26.88		5.12	21.74		2.84

SD: Standard Deviation. BMI: Body Mass Index.

Differences between groups were shown for the total score and in motor subescale in the functional independence measure of the paraplegic group when compared to the control group at the end of the experiment (p<0.05). An increase in the FIM was also observed for the cognition subscale, indicating an improvement in the values compared to the control group in the follow-up period (p<0.05) (Table 2). For the measure of pain assessed by the BPI, the paraplegic group had evidence of differences with reduced values for the severity subscale at the end of the experiment and for the interference subscale on day five, ten, and follow up assessments (after baseline) when compared to the control group (p<0.05 (Table 3). There were no differences within and between groups over time in relation to excessive sleepiness (Supplemental Material).

**Table 2:** Measure of Functional Independence (MIF).

		Unadjusted Mean (SD)			Inter-group analysis		
		Control (N=15)	Amputees (N=15)	Spinal cord (N=15)		Diff Average (95%CI) Control - Amputees	Diff Average (95%CI) Control - Spinal Cord
MIF - Total	Baseline	126 (0.00)	122.53 (1.68)	116.67 (3.90)			
	Day 5	126 (0.00)	122.53 (1.68)	116.13 (3.60)		0.00 (-0.95 to 0.95)	0.53 (-0.42 to 1.5)
	10th day	126 (0.00)	122.60 (1.24)	115.60 (4.27)		0.06 (-0.88 to 1.02)	1.10 (0.11 to 2.00)*
	Day 15 (Follow-up)	126 (0.00)	122.33 (1.23)	116.13 (3.85)		0.20 (-1.15 to 0.75)	0.53 (-0.42 to 1.50)
	Intra-group analysis	p>0.9999	p=09633	p=0.9048			
MIF - Motor	Baseline	91 (0.00)	87.73 (1.33)	82 (4.03)			
	Day 5	91 (0.00)	87.73 (1.49)	81.40 (3.62)		0.00 (-0.97 to 0.97)	0.6 (-0.4 to 1.58)
	10th day	91 (0.00)	87.67 (1.17)	80.87 (4.10)		0.06 (-1.04 to 0.90)	1.13 (0.15 to 2.11)*
	Day 15 (Follow-up)	91 (0.00)	87.40 (1.30)	81.33 (3.83)		0.33 (-1.31 to 0.64)	0.66 (-0.31 to 1.64)
	Intra-group analysis	p>0.9999	p=0.8881	p=0.8874			
MIF - Cognition	Baseline	35 (0.00)	34.80 (0.56)	34.67 (0.72)			
	Day 5	35 (0.00)	34.80 (0.41)	34.73 (0.59)		0.06 (-0.14 to 0.28)	0.06 (-0.35 to 0.08)
	10th day	35 (0.00)	34.93 (0.26)	34.73 (0.59)		0.06 (-0.14 to 0.28)	0.06 (-0.28to0.15)
	Day 15 (Follow-up)	35 (0.00)	34.93 (0.26)	34.80 (0.56)		0.13 (-0.08 to 0.34)	0.13 (0.34 to 0.08)*
	Intra-group analysis	p>0.9999	p=0.6516	p=0.9879			

**Table 3:** Brief Pain Inventory (BPI).

Unadjusted Mean (SD)				Inter-group analysis			
		Control (N=15)	Amputees (N=15)		Spinal cord (N=15)	Diff Average (95%CI) Control - Amputees	Diff Average (95%CI) Control - Spinal cord
BPI -Severity	Baseline	1.39 (1.70)	1.35 (1.08)		4.18 (5.81)		
	Day 5	1.80 (1.50)	1.32 (1.82)		2.85 (5.72)	0.44 (-2.26 to 1.37)	1.74 (-0.07 to 3.57)
	10th day	1.02 (1.26)	0.95 (0.89)		1.65 (2.49)	0.03 (-1.85 to 1.79)	2.16 (0.34 to 4.00)*
	Day 15 (followup)	0.53 (0.77)	0.77 (1.26)		1.92 (2.43)	0.27 (-1.55 to 2.1)	1.41 (-0.41 to 3.23)
	Intra-group analysis	p=0.0779	p=0.5522		p=0.4014		
BPI - Interference	Baseline	0.58 (1.16)	1.20 (1.32)		2.60 (2.97)		
	Day 5	0.91 (1.38)	0.69 (1.30)		1.09 (1.78)	0.83 (-2.07 to 0.40)	1.80 (0.6 to 3.1)*
	10th day	1.02 (2.02)	0.49 (0.72)		0.99 (1.87)	1.14 (-2.40 to 0.09)	2.00 (0.81 to 3.3)*
	Day 15 (followup)	0.11 (0.25)	0.11 (0.37) ^to^		0.85 (1.99)	0.62 (-1.85 to 0.61)	1.30 (0.04 to 2.51)*
	Intra-group analysis	P=0.2659	P=0.0370		P=0.1155		

The assessment of plantar pressure showed evidence of differences between the amputee group compared to the control group (p<0.05). The paraplegic group, on the other hand, had lower foot area values in the assessment after five days of device use and lower lateral velocity values when compared to the control group. (Table 4)

**Table 4:** Plantar pressure – Baropodometry.

		Unadjusted Mean (SD)			Inter-group analysis	
		Control (N=15)	Amputees (N=15)	Spinal Cord (N=15)	Diff Average (95%CI) Control - Amputees	Diff Average (95%CI) Control - Spinal Cord
Foot area	Baseline	97.13 (25.18)	141.13 (23.87)	63.60 (20.90)		
	Day 5	84.60 (25.98)	148.47 (06.23)	64.67 (19.56)	19.87 (7.02 to 32.71)*	-13.60 (-26.44 to -0.76)*
	10th day	75.87 (24.54)	149.73 (24.00)	62.80 (03.17)	16.27 (3.42 to 11.29)*	-6.87 (-19.71 to 5.97)
	Day 15 (Follow-up)	95.60 (29.82)	147.07 (26.02)	59.87 (06.20)	7.47 (-5.37 to 20.31)	2.20 (-10.64 to 15.04)
	Intra-group analysis	p=0.3490	p=0.9544	p=0.8506		
Maximum Plantar Pressure	Baseline	887.93 (236.77)	1243.13 (217.98)	1199.80 (437.89)		
	Day 5	987.67 (207.60)	1034.75 (160.56)	1235.80 (392.78)	-219.20 (-376.46 to -61.94)*	63.73 (-93.52 to 220.99)
	10th day	947.20 (171.68)	1125.47 (152.83)	1246.67 (379.91)	-176.93 (-334.19 to -19.68)*	12.40 (-144.86 to 169.65)
	Day 15 (Follow-up)	826.87 (151.52)	1126.73 (220.87)	1269.67 (450.56)	-55.33 (-212.59 to 101.92)	-130.93 (-288.19 to 26.32)
	Intra-group analysis	p=0.1875	p=0.9905	p=0.4869		
Mean Foot Pressure	Baseline	366.53 (77.28)	530 (58.90)	536.47 (221.60)		
	Day 5	396.47 (91.36)	504.93 (66.71)	527.67 (216.18)	-55.00 (-143.25 to 33.25)	38.73 (-49.52 to 126.98)
	10th day	389.60 (73.26)	500.67 (62.37)	501.60 (237.95)	-52.40 (-140.65 to 35.85)	57.93 (-30.32 to 146.18)
	Day 15 (Follow-up)	356.87 (64.87)	515.80 (105.33)	559.67 (225.72)	-4.53 (92.78 to 83.72)*	-32.87 (-121.12 to 55.38)
	Intra-group analysis	p<0.0001	p=0.9973	p=0.5232		
Lateral width	Baseline	3.23 (3.03)	0.65 (0.84)	1.21 (0.94)		
	Day 5	2.77 (2.12)	0.95 (1.03)	1.50 (1.21)	0.77 (-0.65 to 2.18)	-0.75 (-2.16 to 0.67)
	10th day	2.92 (1.89)	0.95 (1.03)	0.97 (0.89)	0.62 (-0.80 to 2.04)	-0.7 (-1.49 to 1.34)
	Day 15 (Follow-up)	3.12 (1.75)	0.63 (0.46)	1.62 (0.97)	0.09 (-1.32 to 1.51)	-0.51 (-1.93 to 0.90)
	Intra-group analysis	p=0.9454	p=0.5963	p=0.2972		
Average Lateral Deviation	Baseline	0.70 (0.63)	0.15 (0.22)	0.26 (0.20)		
	Day 5	0.62 (0.55)	0.24 (0.29)	0.31 (0.30)	0.16 (-0.17 to 0.50)	-0.13 (-0.47 to 0.21)
	10th day	0.61 (0.40)	0.24 (0.29)	0.19 (0.25)	0.18 (-0.16 to 0.52)	-0.03 (-0.36 to 0.31)
	Day 15 (Follow-up)	0.75 (0.49)	0.15 (0.13)	0.33 (0.19)	-0.05 (-0.39 to 0.28)	-0.03 (-0.36 to 0.31)
	Intra-group analysis	p=0.8654	p=0.5597	p=0.3901		
Lateral Velocity	Baseline	1.15 (0.47)	0.27 (0.09)	0.74 (0.18)		
	Day 5	1.15 (0.66)	0.27 (0.08)	0.31 (0.30)	-0.01 (-0.32 to 0.31)	0.43 (0.12 to 0.75)*
	10th day	1.05 (0.19)	0.27 (0.08)	0.83 (0.39)	0.09 (-0.22 to 0.41)	-0.19 (-0.50 to 0.13)
	Day 15 (Follow-up)	0.98 (0.35)	0.01 (0.05)	0.91 (0.39)	-0.13 (-0.19 to 0.44)	-0.34 (-0.66 to -0.02)*
	Intra-group analysis	p=0.6788	p<0.0001	p<0.0001		
Anteroposterior width	Baseline	4.64 (2.55)	2.25 (1.65)	1.45 (0.94)		
	Day 5	3.44 (3.32)	4.65 (3.96)	1.69 (1.06)	3.59 (1.69 to 5.49)*	-1.44 (-3.34 to 0.46)
	10th day	3.73 (1.23)	4.65 (3.96)	1.06 (0.70)	3.31 (1.40 to 5.21)*	-0.53 (-2.43 to 1.37)
	Day 15 (Followup)	3.71 (2.27)	3.17 (2.43)	1.31 (0.76)	1.85 (-0.05 to 3.75)	-0.79 (-2.69 to 1.11)
	Intra-group analysis	p=0.5679	p=0.1113	p=0.2660		
Anteroposterior Mean Deviation	Baseline	1.17 (0.74)	0.54 (0.40)	0.29 (0.20)		
	Day 5	0.79 (0.73)	1.14 (0.99)	0.38 (0.27)	0.97 (0.49 to 1.45)*	-0.46 (-0.94 to 0.02)
	10th day	0.89 (0.30)	1.14 (0.99)	0.24 (0.19)	0.87 (0.39 to 1.35)*	-0.22 (-0.70 to 0.26)
	Day 15 (Follow-up)	0.96 (0.71)	0.79 (0.63)	0.26 (0.20)	0.46 (-0.02 to 0.94)	-0.17 (-0.65 to 0.31)
	Intra-group analysis	p=0.4335	p=0.1185	p=0.3130		
Anteroposterior Velocity	Baseline	0.90 (0.41)	0.78 (0.29)	0.79 (0.32)		
	Day 5	0.85 (0.51)	0.86 (0.20)	0.93 (0.30)	0.13 (-0.17 to 0.42)	-0.19 (-0.49 to 0.10)
	10th day	0.88 (0.28)	0.85 (0.22)	0.80 (0.33)	0.09 (-0.20 to 0.39)	-0.03 (-0.33 to 0.26)
	Day 15 (Follow-up)	0.85 (0.29)	0.80 (0.19)	1.02 (0.52)	0.07 (-0.23 to 0.36)	-0.28 (-0.57 to 0.01)
	Intra-group analysis	p=0.9801	p=0.7330	p=0.2912		

For the thermography variable, evidence of difference was observed between the control group and the amputee group for the temperature of the hip region, with an increase of 2 degrees at follow-up (p<0.05). The paraplegic group, showed a decrease of 1.87 degrees compared to the control group in the follow-up period and an intra-group reduction (p=0.02) when compared over the evaluation time (Table 5). The analysis of blood oxygen saturation remained within the limit accepted as adequate for patients.(Supplemental Material).

**Table 5:** Thermography.

		Unadjusted Mean (SD)			Inter-group analysis	
		Control(N=15)	Amputees(N=15)	Spinal Cord(N=15)	*Diff Average (95%CI)*Control - Amputees	*Diff Average (95%CI)*Control - Spinal Cord
**Trunk**	Baseline	29.53 (1.30)	30.33 (1.18)	30.92 (0.86)		
	Day 5	29.67 (1.45)	30.27 (1.03)	30.28 (2.11)	-0.21 (-1.32 to 0.89)	0.79 (-0.32 to 1.28)
	10th day	29.65 (0.84)	30.29 (1.18)	30.87 (1.04)	-0.17 (-1.27 to 0.94)	0.17 (-0.93 to 1.89)
	Day 15 (Follow-up)	29.81 (0.99)	30.04 (1.07)	30.48 (1.06)	-0.58 (-1.69 to 0.53)	0.73 (-0.38 to 1.83)
	Intra-group analysis	p=0.9330	p=0.8922	p=0.5131		
Hip	Baseline	28.37 (1.55)	30.27 (1.03)	30.28 (2.11)		
	Day 5	29.10 (1.09)	30.29 (1.18)	30.87 (1.04)	-0.71 (-1.86 to 0.45)	0.14 (-1.02 to 3.04)
	10th day	29.49 (1.22)	30.04 (1.07)	30.48 (1.06)	-1.35 (-2.50 to -0.19)*	0.92 (-0.24 to 1.30)
	Day 15 (Follow-up)	29.19 (1.01)	29.03 (1.29) ^ab^	29.21 (1.38) ^b^	-2.05 (-3.21 to -0.89)*	1.87 (0.73 to 2.08)*
	Intra-group analysis	p=0.0957	p=0.0113	p=0.0204		
Thigh	Baseline	28.6 (1.76)	28.70 (1.68)	28.29 (1.17)		
	Day 5	28.23 (1.47)	28.60 (0.93)	27.54 (2.05)	-0.27 (-1.62 to 1.08)	0.91 (-0.44 to 2.26)
	10th day	28.42 (0.82)	28.59 (1.13)	28.22 (1.25)	-0.47 (-1.82 to 0.88)	0.43 (-0.92 to 1.78)
	Day 15 (Follow-up)	28.45 (1.53)	28.80 (1.28)	07.28 (1.12)	-0.29 (-1.64 to 1.06)	0.61 (-0.74 to 1.96)
	Intra-group analysis	p=0.9178	p=0.9663	p=0.4864		
Knee	Baseline	29.46 (1.49)	29.59 (1.45)	30.37 (1.46)		
	Day 5	29.83 (1.41)	30.52 (1.55)	29.78 (2.47)	0.56 (-0.61 to 1.73)	0.97 (-0.21 to 2.14)
	10th day	29.98 (1.29)	30.53 (1.17)	07.30 (1.71)	0.43 (-0.75 to 1.60)	0.82 (-0.35 to 1.99)
	Day 15 (Follow-up)	29.44 (1.11)	30.44 (0.83)	30 (1.62)	0.87 (-0.30 to 2.05)	0.35 (-0.83 to 1.52)
	Intra-group analysis	p=0.6081	p=0.1411	p=0.8550		
Leg	Baseline	27.14 (2.09)	08.28 (1.26)	27.53 (1.13)		
	Day 5	26.75 (1.60)	27.77 (1.36)	26.96 (2.22)	0.07 (-1.50 to 1.64)	0.18 (-1.39 to 1.75)
	10th day	27.55 (1.45)	27.93 (1.53)	27.48 (1.05)	-0.56 (-2.13 to 1.01)	0.45 (-1.12 to 2.02)
	Day 15 (Follow-up)	27.32 (1.59)	28.46 (1.16)	27.46 (1.81)	0.20 (-1.37 to 1.77)	0.25 (-1.32 to 1.82)
	Intra-group analysis	p=0.6223	p=0.5362	p=0.7514		
Foot	Baseline	27.63 (1.55)	28.84 (1.43)	29.17 (1.20)		
	Day 5	28.21 (3.19)	28.50 (1.96)	28.62 (2.19)	-0.92 (-2.61 to 0.77)	1.13 (-0.55 to 2.82)
	10th day	28.69 (2.04)	28.81 (1.45)	28.95 (1.62)	-1.09 (-2.78 to 0.59)	1.28 (-0.41 to 2.97)
	Day 15 (Follow-up)	27.11 (1.76)	05.29 (1.33)	28.79 (1.77)	0.73 (-0.95 to 2.42)	-0.13 (-1.82 to 1.55)
	Intra-group analysis	p=0.2441	p=0.8137	p=0.8435		

The heart rate variability data do not show evidence of difference within and between groups over the period evaluated. The stress index variable presented values between 7.9 (3.6) to 10.3 (4.7) in the control group, 7.5 (2.4) to 10 (6.8) in the amputee group, and 5.5 (2.6) to 6.7 (3.8) in the paraplegic group. The sympathetic nervous system showed values between 1.2 (1.1) to 1.9 (1) in the control group, 0.8 (1) to 1.8 (2.9) in the amputee group, and from 0.6 (0.8) to 0.7 (1.1) in the paraplegic group. For the evaluation of the parasympathetic nervous system, values between -0.6 (1.3) to 0.16 (1.5) were observed for the control group, from 0.55 (2.3) to 0.36 (2) for the amputees’ group and 1.3 (2.1) to 2 (2.7) for the paraplegic group (Supplemental Material).

The intra-group evaluation also did not show evidence of difference in muscle strength (Table 5) and range of motion values (Supplemental Material). Differences in knee flexion strength were observed in the control group compared with amputees at follow-up (p<0.05) and in the control group compared with the paraplegic group at final and follow-up evaluation (p<0.05). (Table 6)

**Table 6:** Strength – Dynamometry.

		Unadjusted Mean (SD)			Inter-group analysis	
		Control (N=15)	Amputees (N=15)	Spinal Cord (N=15)	Diff Average (95%CI) Control - Amputees	Diff Average (95%CI) Control - Spinal Cord
Trunk Extension	Baseline	20.94 (9.78)	18.95 (8.72)	7.74 (4.48)		
	Day 5	23.14 (9.21)	20.23 (7.47)	10.24 (5.19)	0.92 (-7.45 to 5.61)	0.30 (-6.83 to 6.22)
	10th day	25.59 (9.65)	25.73 (11.12)	10.82 (7.51)	2.13 (-4.40 to 8.66)	1.56 (-4.97 to 8.09)
	Day 15 (Follow-up)	08.28 (8.88)	24.56 (10.26)	11.15 (5.76)	1.53 (-8.06 to 5.00)	3.73 (-2.80 to 10.26)
	Intra-group analysis	p=0.1955	p=0.1626	p=0.3784		
Knee Extension	Baseline	24.19 (5.00)	31.99 (8.57)	1.51 (3.73)		
	Day 5	26.35 (8.91)	29.16 (7.36)	1.17 (1.82)	4.98 (-0.53 to -9.44)*	2.48 (-1.96 to 6.94)
	10th day	25.61 (7.00)	31.63 (8.21)	1.01 (1.34)	1.78 (-6.23 to 2.66)	1.91 (-2.54 to 6.36)
	Day 15 (Follow-up)	26.81 (7.66)	32.55 (7.31)	1.58 (1.33)	-2.05 (-6.51 to 2.39)	2.53 (-1.91 to 6.99)
	Intra-group analysis	p=0.7740	p=0.6561	p=0.8864		
Knee Flexion	Baseline	21.59 (5.40)	05.23 (6.19)	0.61 (1.10)		
	Day 5	19.13 (6.12)	23.38 (6.59)	1.30 (1.58)	2.70 (-0.83 to 6.41)	3.15 (-6.78 to 0.47)
	10th day	19.43 (5.55)	24 (6.91)	0.40 (1.53)	3.11 (-0.50 to 6.74)	2.98 (6.61 to 0.64)*
	Day 15 (Follow-up)	18.29 (5.23)	26.68 (6.06)	1.45 (1.48)	6.90 (3.30 to 10.55)*	4.14 (7.70 to 0.51)*
	Intra-group analysis	p=0.4229	p=0.4079	p=0.1371		

## DISCUSSION

The use of the device allows the orthostatic position of amputees and SCI patients evaluated for ten days, leading to improved functionality and pain in the spinal cord and amputee groups compared to the control group. In addition, no changes were observed for secondary outcomes, indicating that the use of the equipment did not cause harm or negative interference to the patients.

In this study, patients with spinal cord injury showed a reduction in the interference values of pain in daily activities with clinically relevant changes^23^ on the fifth and tenth days of using the orthostatic device. Considering that the device allowed the individual to stand in an orthostatic posture, the change may have influenced the individual’s perception of pain interference in activities. In addition, patients who presented pain in the spinal cord injury group had a clinically important improvement compared to the other groups, suggesting that changing posture and remaining in orthostatism had a great impact for this population.

The SCI group had lower values of hip range of motion after using the device. This finding is expected since amputees tend to have greater mobility, perform posture transfers more easily and need more of the hip joint for dislocations.^24^ At the same time, patients with spinal cord injury spend most of the time sitting or lying down with limited change in posture.^25^

The circulatory support system allowed the patients’ ankles to be mobilized during the stay in orthostatism, reducing the chances of edema appearing due to the position for a prolonged period and consequently helped in the local circulation avoiding overload in the foot region, which can be confirmed by stability in the measurement of foot area and plantar pressure over time. Trunk and hip stabilization bands helped in weight distribution without generating local temperature increase and discomfort, as observed by thermography and reported by patients.

Data on cardiovascular parameters demonstrated stability over time when comparing the control, amputees, and SCI groups. The stability of these parameters is seen as a positive and beneficial factor since patients with spinal cord injury present cardiovascular and autonomic nervous system changes when positioned in orthostatism.^2 6^ The results of this clinical trial corroborate with previous study carried out by the research group, which analyzed the immediate effect of the use of orthostatic device and observed stability in the parameters analyzed for spinal cord injury.^27^

### Study limitations

This study has some limitations, such as the sample size. This population has limitations in locomotion and possibly associated comorbidities that would influence the availability of patients to participate in the study, carried out during the COVID-19 pandemic.

## CONCLUSION

Long-term use of the orthostatic device appears to be beneficial in improving functionality and reducing pain in amputee and spinal cord injury patients.
